# Supplementary material for: Enhancing Physicochemical Properties and Single Cell Performance of Sulfonated Poly(arylene ether) (SPAE) Membrane by Incorporation of Phosphotungstic Acid and Graphene Oxide: A Potential Electrolyte for Proton Exchange Membrane Fuel Cells
Source: Polymers (Basel). 2021 Jul 19;13(14):2364. doi: 10.3390/polym13142364 (PMC8309513; doi:10.3390/polym13142364)
Supplement: Supplementary file 1 [file polymers-13-02364-s001.zip › polymers-1257961-supplementary.pdf]

polymers

MDPI

Supporting Material

Enhancing Physicochemical Properties and Single Cell Performance of Sulfonated Poly (Arylene Ether) (SPAE) Membrane by Incorporation of Phosphotungstic Acid and Graphene Oxide: A Potential Electrolyte for Proton Exchange Membrane Fuel Cells

Sung Kwan Ryu [1], Ae Rhan Kim [1,2,*], Mohanraj Vinothkannan [3], Kyu Ha Lee [2], Ji Young Chu [2] and Dong Jin Yoo [1,2,3,*]

1 Department of Energy Storage/Conversion Engineering of Graduate School (BK21 FOUR), Hydrogen and Fuel Cell Research Center, Jeonbuk National University, Jeonju, Jeollabuk-do 54896, Republic of Korea; hellomong35@naver.com
2 Department of Life Science, Jeonbuk National University, Jeonju, Jeollabuk-do 54896, Republic of Korea; carumiss@naver.com (K.H.L.); ebbuneg@hanmail.net (J.Y.C.)
3 R&D Education center for whole life cycle R&D of fuel cell systems, Jeonbuk National University, Jeonju, Jeollabuk-do 54896, Republic of Korea; vinothkannanram@gmail.com
* Correspondence: kimaerhan@jbnu.ac.kr (A.R.K.); djyoo@jbnu.ac.kr (D.J.Y.)

Citation: Ryu, S.K.; Kim, A.R.; Vinothkannan, M.; Lee, K.H.; Chu, J.Y.; Yoo, D.J. Enhancing Physicochemical Properties and Single Cell Performance of Sulfonated Poly (Arylene Ether) (SPAE) Membrane by Incorporation of Phosphotungstic Acid and Graphene Oxide: A Potential Electrolyte for Proton Exchange Membrane Fuel Cells. *Polymers* **2021**, *13*, x. https://doi.org/10.3390/xxxxx

Academic Editor(s): Alessandra Carbone

Received: 28 May 2021
Accepted: 12 July 2021
Published: date

Publisher's Note: MDPI stays neutral with regard to jurisdictional claims in published maps and institutional affiliations.

Copyright: © 2021 by the authors. Submitted for possible open access publication under the terms and conditions of the Creative Commons Attribution (CC BY) license (http://creativecommons.org/licenses/by/4.0/).

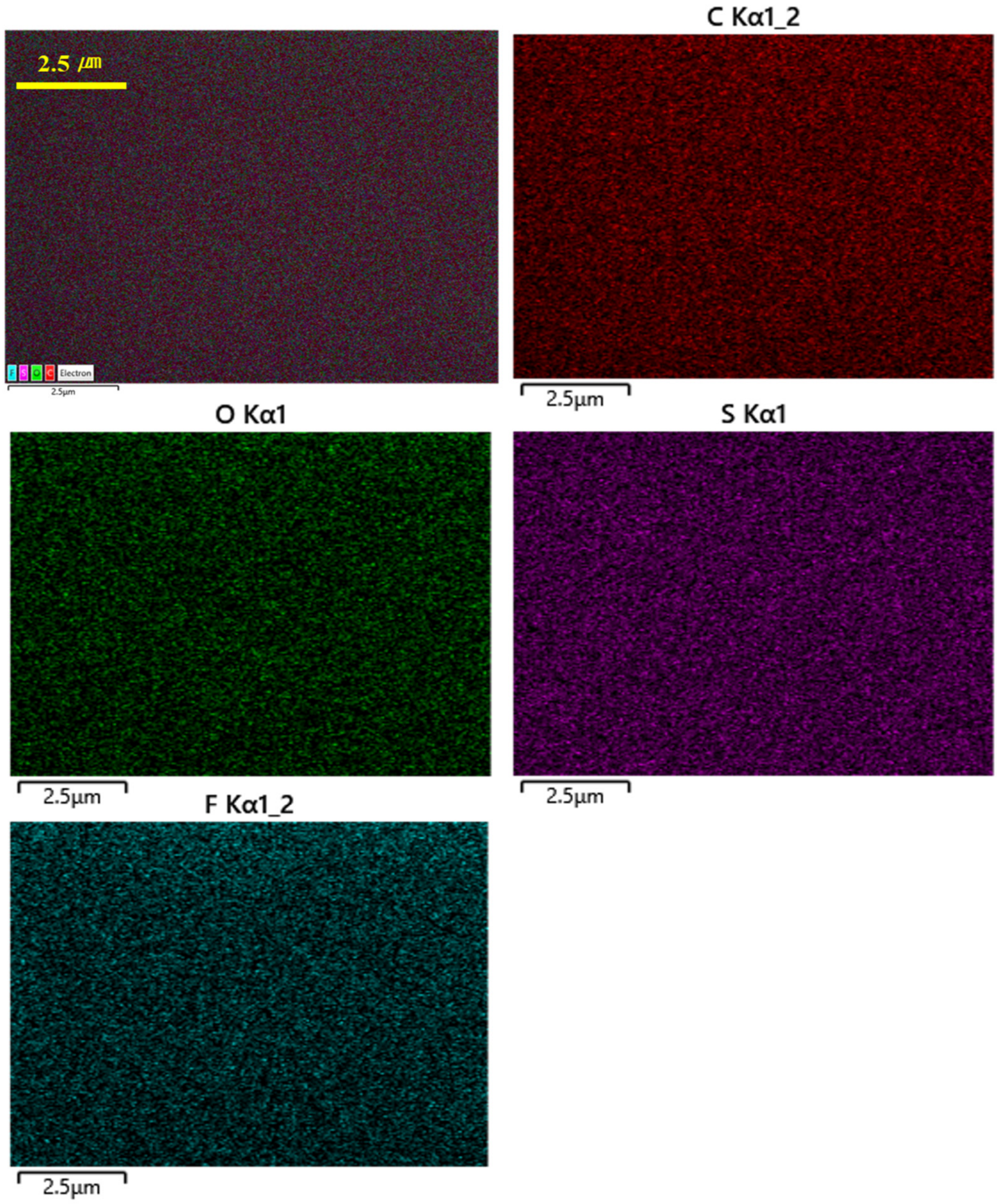

Figure S1. EDAX elemental mappings of bare SPAE membrane.

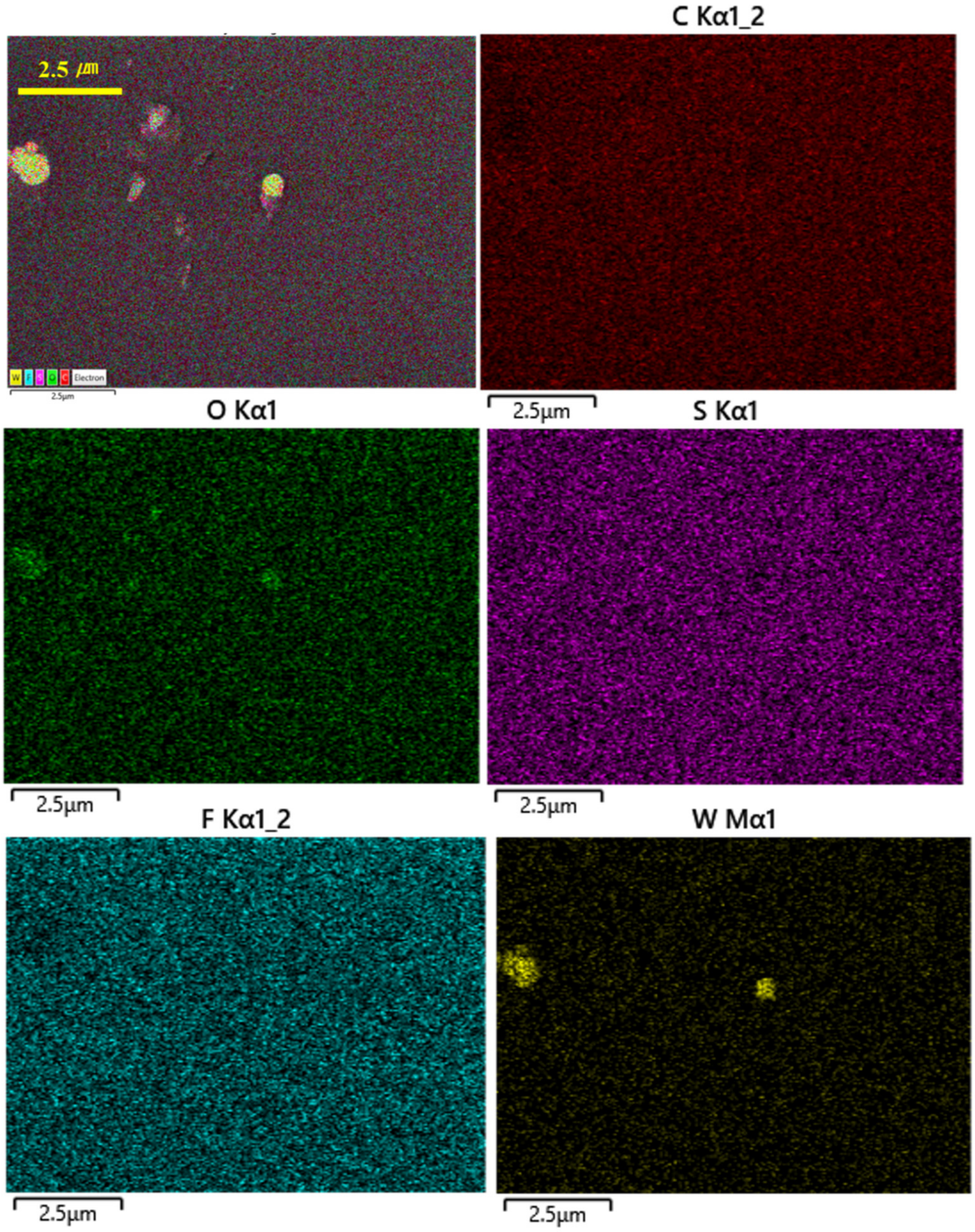

Figure S2. EDAX elemental mappings of SPAE/GO/PWA (36 wt%) membrane.

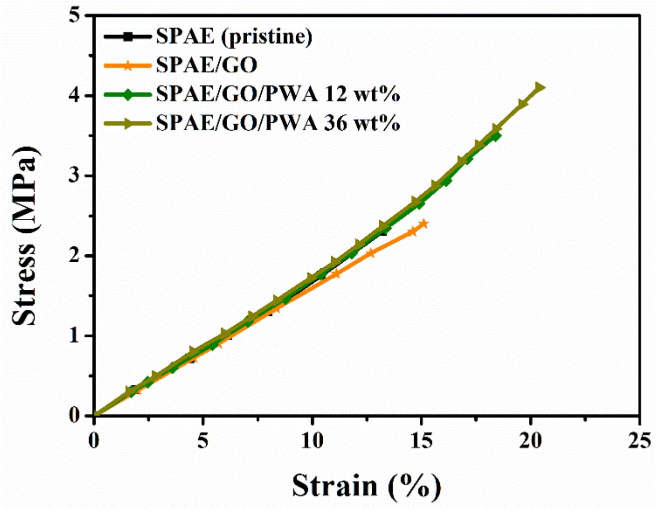

Figure S3. Stress-strain curves of bare SPAE and composite membranes.

Table S1. IEC, water uptake, swelling ratio, and proton conductivity of bare SPAE and SPAE/GO/PWA composite membranes.

Membrane	IEC (mequiv g^{-1})	Water Uptake (%)		Swelling Ratio (%)		Proton Conductivity (mS cm^{-1})	
		30 °C	90 °C	30 °C	90 °C	30 °C	90 °C
SPAE (pristine)	1.68	3	21	6	8	51.2	104.1
SPAE/GO	1.69	4	23	6	9	52.2	106.6
SPAE/GO/PWA 1 wt%	1.72	7	26	7	10	56	113.5
SPAE/GO/PWA 3 wt%	1.78	10	30	8	11	61.4	125.1
SPAE/GO/PWA 6 wt%	1.84	14	36	10	13	66.1	139.1
SPAE/GO/PWA 12 wt%	1.89	20	43	12	16	77.7	154.7
SPAE/GO/PWA 24 wt%	1.95	24	46	13	19	91.3	168.7
SPAE/GO/PWA 36 wt%	2.02	27	52	15	22	107.3	186.3
